# The human Shu complex functions with PDS5B and SPIDR to promote homologous recombination

**DOI:** 10.1093/nar/gkz738

**Published:** 2019-09-06

**Authors:** Julieta Martino, Gregory J Brunette, Jonathan Barroso-González, Tatiana N Moiseeva, Chelsea M Smith, Christopher J Bakkenist, Roderick J O’Sullivan, Kara A Bernstein

**Affiliations:** 1 Department of Microbiology and Molecular Genetics, UPMC Hillman Cancer Center, University of Pittsburgh School of Medicine, Pittsburgh, PA 15213, USA; 2 Department of Pharmacology and Chemical Biology; UPMC Hillman Cancer Center, University of Pittsburgh School of Medicine, Pittsburgh, PA 15213, USA; 3 Department of Radiation Oncology, UPMC Hillman Cancer Center, University of Pittsburgh School of Medicine, Pittsburgh, PA 15213, USA

## Abstract

RAD51 plays a central role in homologous recombination during double-strand break repair and in replication fork dynamics. Misregulation of RAD51 is associated with genetic instability and cancer. RAD51 is regulated by many accessory proteins including the highly conserved Shu complex. Here, we report the function of the human Shu complex during replication to regulate RAD51 recruitment to DNA repair foci and, secondly, during replication fork restart following replication fork stalling. Deletion of the Shu complex members, *SWS1* and *SWSAP1*, using CRISPR/Cas9, renders cells specifically sensitive to the replication fork stalling and collapse caused by methyl methanesulfonate and mitomycin C exposure, a delayed and reduced RAD51 response, and fewer sister chromatid exchanges. Our additional analysis identified SPIDR and PDS5B as novel Shu complex interacting partners and genetically function in the same pathway upon DNA damage. Collectively, our study uncovers a protein complex, which consists of SWS1, SWSAP1, SPIDR and PDS5B, involved in DNA repair and provides insight into Shu complex function and composition.

## INTRODUCTION

DNA double-strand breaks (DSBs) are among the most cytotoxic DNA lesions and their misrepair can result in genetic instability. Homologous recombination (HR) is primarily used to repair DSBs during the S and G2 cell cycle phases, where a sister chromatid or homologous chromosome is used as a repair template (1). During HR, the DSB end is resected and coated with the single-stranded DNA binding complex RPA. RAD51 then displaces RPA forming a nucleoprotein filament, which is required for the homology search and strand invasion steps that define HR. As such, RAD51 filament formation is highly regulated by a group of proteins collectively called the RAD51 mediators, which include BRCA2, PALB2, the RAD51 paralogs and the Shu complex (2,3).

Among this diverse group of RAD51 mediators are the canonical RAD51 paralogs (RAD51B, RAD51C, RAD51D, XRCC2 and XRCC3), proteins that share amino acid similarity to RAD51 but whose functions have remained elusive due to their embryonic lethality in mice (4–7), protein insolubility and low abundance (8). Not surprisingly, germline and somatic mutations in RAD51 and its mediators have been extensively linked to genetic instability, cancer predisposition and hereditary diseases such as Fanconi anemia (2,9–11).

Recently, a novel RAD51 paralog-containing complex, the Shu complex, was discovered in budding yeast (12) and shown to stimulate RAD51 loading onto RPA-coated ssDNA (13). Although the Shu complex is conserved in all eukaryotic lineages, including humans (14–17), both the precise function and composition of the human Shu complex have remained elusive. The human Shu complex is composed of SWSAP1, a newly identified RAD51 paralog (15), and SWS1, a SWIM domain-containing Shu2/SWS1 protein family member (14,16). In yeast and worms, Shu complex disruption results in increased mutagenicity and impaired homologous recombination (12,14,17–20). Unlike other RAD51 mediators, disruption of the Shu genes primarily results in MMS sensitivity and not to other DNA damaging agents such as ionizing radiation, hydroxyurea (which depletes dNTP pools), or the TOPO2 inhibitor etoposide (12,21). Consistent with a conserved function for the human Shu complex, *SWS1* and *SWSAP1* siRNA depletion in human cells results in increased MMS sensitivity and reduced RAD51 foci upon MMS exposure (14,15). Increasing evidence from yeast demonstrates that the Shu complex facilitates tolerance of MMS-induced DNA damage by promoting HR during replication (21). The role of the human Shu complex in promoting RAD51 in a replicative context and whether human Shu complex disrupted cells are primarily MMS sensitive remains unknown. Furthermore, the yeast Shu complex is a heterotetramer and therefore it likely that additional mammalian Shu complex members exist.

In this study, we used CRISPR-Cas9 to knock out human Shu complex members, *SWS1* and *SWSAP1*, in a non-tumorigenic human cell line (RPE-1) and assessed the phenotypes associated with Shu complex loss. We find that sgSWS1 and sgSWSAP1 cells are primarily sensitive to MMS and mitomycin C [MMC, which creates interstrand crosslinks (ICL)]. Upon DNA damage, sgSWS1 and sgSWSAP1 cells exhibit a delayed and reduced RAD51 foci response and fewer sister chromatid exchanges. Upon replication fork stalling, sgSWS1 and sgSWSAP1 cells have defects in replication fork restart. Additionally, to find additional Shu complex binding partners, we performed a BioID screen and identified SPIDR and PDS5B as two novel Shu complex binding partners. We find that SPIDR and PDS5B both physically interact as well as genetically function in the same pathway upon MMS damage. Together, our findings reveal novel roles for the human Shu complex to promote RAD51-mediated HR upon replicative damage and novel complex members.

## MATERIALS AND METHODS

### Cell culture

RPE-1 cells, hTERT-immortalized retinal epithelial cells (ATCC CRL-4000), were used for all experiments. Cells were maintained as a sub-confluent monolayer in DMEM/F-12 (Gibco 11330-032) supplemented with 10% FBS (VWR Seradigm 1500-500) and 0.01 mg/ml Hygromycin B (Invitrogen 10687010). Cells were passaged every 3 days and routinely tested for mycoplasma contamination using a PCR-based assay (Sigma MP0025). Cell lines were authenticated using STR profiling at the University of Arizona Genetics Core. All experiments were maintained in a humidified 5% CO_2_ incubator at 37°C. Cell culture plates, dishes and flasks were purchased from Thermo Scientific (BioLite). PBS was purchased from Corning (21-040-CV) and 0.25% Trypsin-EDTA from Gibco (25200-056).

### Generation of SWS1 and SWSAP1 knockout cell lines by CRISPR

pLentiCRISPR v2 plasmids (22) encoding sgRNAs against SWS1 (gRNA sequence: TGATGGACTGTCGATCAACT) or SWSAP1 (gRNA sequence: CTAGAGTCGTTCCGGTCCCG) were purchased from GenScript. The plasmids were packaged in lentivirus by the Genome Editing, Transgenic and Virus (GETV) Core Facility at Magee Women's Research Institute using the FUGW lentiviral vector backbone (23). Cells grown in 6-well plates were transduced with a MOI = 5 and 8 ug/ml of polybrene for 12 h for two consecutive nights. Cells were passaged and briefly placed under selective pressure using 20 ug/ml of puromycin (Mirus 5940). Cells were diluted and plated on 96-well plates at single cell density. Wells with a single colony were selected for further expansion. Genomic DNA was extracted from a subset of clones using the Quick-gDNA MidiPrep Kit (Zymo Research D3100). A region of ∼300 bp around the sgRNA target area of SWS1 or SWSAP1 was PCR-amplified using Phusion PCR Master Mix (Thermo Scientific F531S). Primers for genomic DNA amplification were as follows: AAGTCTAGTGATCCTTTGGGCA and AAAGCCTTTTAACAGTCCAGGAA (SWS1 gene), and TGCTCGGTACACCAGGATCT and TTGCTAACACCGCCCATCAT (SWSAP1 gene). In order to sequence each allele individually, PCR products were gel purified with the NucleoSpin Kit (Macherey-Nagel 740609) and cloned into pCR4Blunt-TOPO plasmid using a kit (Invitrogen 450159). Bacteria were transformed with the TOPO reaction and grown overnight on selective agar plates. Ten bacterial colonies per clone were grown overnight and plasmid DNA was mini-prepped using a kit (Qiagen 27106). Plasmids were sequenced using generic T7 and T3 promoter primers (Genewiz). For experiments, two SWS1 or SWSAP1 clones were selected based on the presence of genetic modifications that induced early stop codons. CRISPR cell lines were maintained as described for the parental RPE-1 cell line.

### Generation of BioID cell lines and BioID pulldown

RPE-1 cells were transfected with pcDNA3.1 mycBioID empty vector or containing SWS1 or SWSAP1 cDNA using TransIT-LT1 reagent (Mirus MIR 2304) at a 1:2 ratio of plasmid to transfection reagent. Cells were selected with 800 ug/ml G418 (Gibco 10131035) and expanded. A total of 60 million cells of RPE-1-myc-BirA-empty, RPE-1-myc-BirA-SWS1 and RPE-1-myc-BirA-SWSAP1 were incubated with 50 uM biotin (Sigma B4501) for 24 h. During the last 5 h 0.5 mM MMS was added for 1 h and cells were left to recover for 4 h. Cells were harvested following the protocol by Roux *et al.* (24). Cells were lysed with lysis buffer (50 mM Tris–Cl pH 8.5, 500 mM NaCl, 0.2% SDS, 2% Triton X-100) and sonicated until lysate was clear. Lysates were diluted with 50 mM Tris–Cl pH 7.4, sonicated again and centrifuged. Supernatants were mixed with streptavidin beads (Invitrogen 65001) and incubated overnight at 4°C. After extensive washing (24), beads were washed with an additional buffer (50 mM Tris–Cl pH 7.4, 50 mM NaCl) and resuspended in a 1:3 solution of LDS 4× sample buffer (Invitrogen NP0007) and above buffer with 2% SDS. Protein was eluted from beads by boiling 5 min at 98°C. Two independent experiments were done.

### Mass spectrometry analysis of BioID samples

Elutes (25 ul) were analyzed using liquid chromatography/mass spectrometry/mass spectrometry (LC/MS/MS) by MS Bioworks (Ann Arbor, MI, USA). Samples were separated on a 10% Bis-Tris gel, followed by Coomassie stain and excision of lanes into ten pieces. Gel pieces were processed as follows: 25 mM ammonium bicarbonate wash was followed by an acetonitrile wash, subsequently samples were reduced at 60°C with 10 mM dithiothreitol and alkylated at RT with 50 mM iodoacetamide. Trypsin digest was conducted at 37°C for 4 h and followed by formic acid quenching. The supernatant was directly analyzed without processing.

Nano LC/MS/MS with a Waters NanoAcquity HPLC system/ThermoFisher Q Exactive was used to analyze the gel digests. A trapping column was used to load peptides and eluted at 350 nl/min on a 75 μm analytical column. The columns used Jupiter Proteo resin (Phenomenex). The mass spectrometer with MS and MS/MS performed in the Orbitrap at 70 000 FWHM and 17 500 FWHM resolutions, respectively, operating in data-dependent mode. The fifteen most abundant ions were selected for MS/MS. Data were analyzed using Mascot and the files were validated using the Scaffold software, which enabled filtering and creation of a non-redundant sample list. Data required a minimum of two unique protein peptides and were filtered at 1% protein and peptide level false discovery rate (FDR).

### Antibodies

The following primary antibodies were used in western blotting: EMSY (1:500, abcam 32329), FLAG (1:1000, Sigma F3165), HA (1:1000, Santa Cruz Biotechnology sc-895), myc (1:1000, Santa Cruz Biotechnology sc-40), APRIN/PDS5B (1:500, Novus NB100-755), SPIDR (1:250, Sigma HPA041582), Streptavidin (1:1000, LI-COR 925-68079), and α-Tubulin (1:1000, Cell Signaling 3873). The following primary antibodies were used in immunofluorescence: RAD51 (1:200, Santa Cruz Biotechnology sc-8349), RPA32 (1:250, abcam 170190); and fiber analysis: rat anti-BrdU (1:50, abcam 6326), mouse anti-BrdU (1:100, BD Biosciences 347580). Secondary antibodies and antibodies conjugated to beads used in immunoprecipitations are listed under their corresponding experimental descriptions.

### DNA damaging agents

Methyl methane sulfonate (MMS) (Sigma 129925) stocks were made fresh with diH_2_0. Cisplatin (Sigma P4394) was resuspended in water (3.3 mM) and stocks stored at −20°C. Mitomycin C (MMC) (Sigma M4287) was resuspended in water (6 mM) and stocks stored at 4°C. Hydroxyurea (HU) (Sigma H8627) stocks were made fresh with diH_2_O. Compounds were protected from light when required. Ionizing radiation was performed using a Nordion Cs137 Gamma Cell 1000D. All other general laboratory reagents were purchased from Sigma unless otherwise indicated.

### Plasmids

pcDNA3-3HA-SWS1 (Martin *et al.* 2006) and pDONR201-myc-SWSAP1 (15) were generously provided by Dr. Paul Russell (The Scripps Research Institute) and Dr. Jun Huang (Zhejiang University), respectively. The plasmid pcDNA3.1-mycBioID (24) was obtained from Addgene (#35700). For BioID, SWS1 and SWSAP1 cDNA was PCR-amplified from the pcDNA3-3HA-SWS1 and pDONR201-MYC-SWSAP1 vectors and sub-cloned into pcDNA3.1-mycBioID, using restriction enzyme cloning (Supplemental Table S1). pCMV3-FLAG-SPIDR was purchased from Sino Biological Inc. pCMV-2B (FLAG)-PDS5B was synthesized by Genewiz. pcDNA3.1-EMSY (25) was generously provided by Dr. Douglas Levine (New York University). Before transfection into mammalian cells, all plasmids were maxi-prepped (Qiagen 12163). For use in yeast-two and -three-hybrid experiments, PDS5B, SPIDR, SWS1 and SWSAP1 cDNA was PCR-amplified from the above vectors (Supplemental Table S1). The FIGNL1 cDNA was purchased from Sino Biologicals (HG15206-G). All cDNAs were sub-cloned into pGAD, pGBD (26) and pRS-ADH416 yeast plasmids using restriction enzyme digest (Supplemental Table S1). Yeast plasmids containing SWS1 or SWSAP1 were previously described (16,17). All plasmid inserts were sequenced verified.

### Clonogenic survival

**Figure 1.**
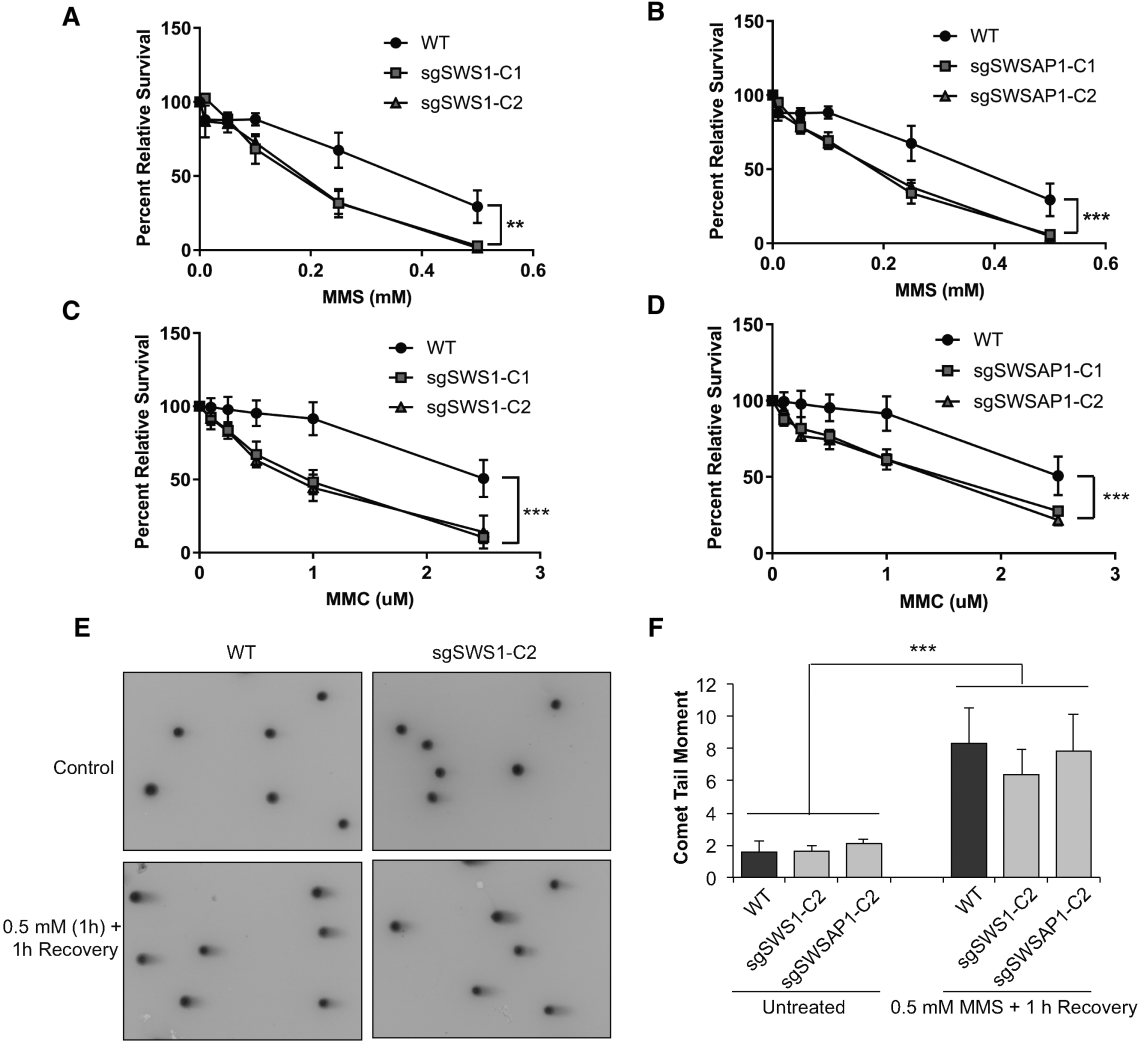
sgSWS1 and sgSWSAP1 RPE-1 cells are MMS and MMC sensitive. (**A**) Clonogenic survival assays of WT parental RPE-1, and two independent sgSWS1 RPE-1 clones (sgSWS1-C1, and sgSWS1-C2) upon MMS exposure (*P* = 0.0005). All cells were treated with the indicated MMS concentrations for 1 h, seeded at colony forming density and stained with crystal violet 8 days post-seeding. Mean percent survival relative to parental RPE-1 cells and standard error of the mean are shown. All experiments were performed in triplicate. Statistical differences were calculated by fitting a non-linear regression curve to each data set and comparing slopes between WT and sgSWS1 cells. (**B**) Same as (A) except that two independent sgSWSAP1 clones (sgSWSAP1-C1 and sgSWSAP1-C2) were compared to WT. (**C**) Clonogenic survival assays of WT parental RPE-1, and two independent sgSWS1 RPE-1 clones (sgSWS1-C1, and sgSWS1-C2) upon MMC exposure (1 h) (*P*< 0.0001). Survival was performed and analyzed as explained in (A). (**D**) Same as (C) except that two independent sgSWSAP1 clones (sgSWSAP1-C1 and sgSWSAP1-C2) were compared to WT. (**E**) Representative images of comet assay after MMS exposure (0.5 mM for 1 h and 1 h recovery) in WT and sgSWS1-C2 RPE-1 cells. (**F**) DSBs were measured by determining the neutral comet tail moment in WT, sgSWS1-C2, and sgSWSAP1-C2 RPE-1 cells after exposure to 0.5 mM MMS for 1 h followed by a 1 h recovery. Experiments were performed in triplicate with 50–100 comets analyzed per experiment. Data are expressed as mean comet tail moment with standard error of the means graphed. Statistical significance (***P< 0.0005) was calculated using a two-way ANOVA (cell line and treatment as factors) followed by a Tukey test.

Cells were seeded in 6 well plates and treated as indicated in Figure 1 and Supplementary Figure SS2A. Following indicated treatment, cells were trypsinized, counted using a Z1 Coulter Counter (Beckman Coulter), centrifuged and serially diluted. A total of 860 cells were resuspended in 12 ml of media and three 60 mm dishes were seeded with 3 ml of cells each. Colonies were allowed to grow for 8 days. For cell staining, dishes were rinsed 2× with PBS, fixed in methanol for 20 min and stained with crystal violet solution (0.5% crystal violet, 20% methanol) for 30 min. Colonies were manually counted. Results are expressed as percent relative survival compared to untreated controls. A total of three-five independent experiments were performed per cell lines.

### Co-immunoprecipitation

Cells were seeded in 100 mm dishes, grown to 70% confluency, and transfected using a 1:2 ratio of DNA to transfection reagent (TranslT-LT1 reagent, Mirus MIR 2304). After 72 h of incubation, cells were trypsinized, centrifuged and pellets lysed with 250 ul of IP lysis buffer (50 mM Tris–HCl pH 7.5, 100 mM NaCl, 2 mM MgCl_2_, 10% glycerol, 0.1% NP-40) for 20 min on ice. Benzonase nuclease was added (Millipore 70746) to digest DNA. All lysis buffers were supplemented with 1× protease inhibitor cocktail (Roche 1187358001) and 1 mM PMSF (Sigma 78830). After a 20 min spin at maximum speed, lysates were mixed with 50 ul of Flag- (Sigma A2220), HA- (Santa Cruz Biotechnology sc-7392 AC) or Myc-conjugated agarose beads (Santa Cruz Biotechnology sc-40 AC), and incubated on a rotator overnight at 4°C. After washing beads 3x/10 min in lysis buffer, beads were gently spun down, mixed 1:1 with 2× Laemmli sample buffer (65.8 mM Tris–HCl pH 6.8, 26.3% glycerol, 2.1% SDS, 0.01% bromophenol blue, 100 mM DTT) and boiled at 99°C for 5 min.

### DNA fiber spreading

Cells were pulsed with 20 uM IdU and 200 uM CldU for 20 min each. For fork protection experiments, 4 mM HU was added after the pulses for 5 h. For fork restart experiments, 2 mM HU was added for 2 h in between the IdU and CldU pulses. Cells were harvested by trypsinization and 2000 cells were placed on a microscopy slide and lysed with 200 mM Tris–HCl pH 7.4, 50 mM EDTA, 0.5% SDS for 6 min. Slides were tilted to 15–20° to allow for fiber spreading. After drying, DNA was fixed with 3:1 methanol: acetic acid for 5 min, and denatured with 2.5 N HCl for 1 h. Slides were blocked with 10% goat serum/0.1% Triton X-100 in PBS for 1 h. All antibodies were diluted in blocking buffer, incubated at 37°C, and washed 3×/5 min with PBS-0.05% Tween-20. First, slides were incubated with anti-BrdU antibodies for 1 h, followed by Alexa Fluor 488 anti-rat and Alexa Fluor 555 anti-mouse IgG1 for 30 min. Slides were dried, coverslipped and images were taken with a Nikon Eclipse Ti-E epifluorescence microscope equipped with a 100× objective (Plan Apo, 100× 1.45, oil), a Photometrics CoolSnapHQ2 camera and appropriate filters (GFP and Texas Red). Experiments were done in duplicate and a total of 50–100 fibers were analyzed per replicate.

### FACS

Cells were treated as needed, harvested with trypsin, spun down and pellets were prepared using Click-iT EdU Alexa Fluor™ 488 Flow Cytometry Assay Kit (Invitrogen C10425) following the manufacturer's instructions. After the click reaction, cells were incubated with FxCycle Far Red Stain (Invitrogen F10348) and RNase A (20 mg/ml) for 30 min on ice. Cells were sorted at the Cytometry Facility (UPMC Hillman Cancer Center) using a Becton Dickinson (Accuri) C6 instrument. Analysis was performed using BD Accuri C6 Software.

### Immunofluorescence

Cells were seeded in 35 mm glass bottom dishes (MatTek P35G-1.5-14-C) and treated as needed. At the end of treatment, cells were washed with PBS, and pre-extracted for 1 min with extraction buffer (50 mM PIPES pH 6.9, 25 mM KCl, 3 mM EGTA, 1 mM MgSO_4_, 0.5% Triton X-100), followed by fixation in 4% paraformaldehyde in PBS (Alfa Aesar J61899) for 20 min at 4°C. Cells were washed 2× with PBS, permeabilized with 0.5% Triton-X100 in PBS for 10 min at RT, washed 2× with PBS and blocked for 1 h at RT (1% BSA, 10% goat serum in PBS). Primary antibodies were incubated overnight at 4°C in 1% BSA in PBS. Cells were washed 3x/5min with PBS, and incubated with secondary antibodies (1:2000, Invitrogen goat anti-mouse or anti-rabbit IgG (H+L) Alexa Fluor 488) in 1% BSA in PBS for 1 h at RT. After washing with PBS and drying, coverslips were mounted with Prolong Gold Antifade with DAPI (Invitrogen P36935). At least 50 cells were analyzed per condition. Images were taken with a Nikon Eclipse Ti-E epifluorescence microscope equipped with a 100x objective (Plan Apo, 100× 1.45, oil), a Photometrics CoolSnapHQ2 camera and appropriate filters (DAPI, GFP, Texas Red and Cy5). The numbers of foci per cell were quantified using Nikon Elements Software. Experiments were repeated in triplicate.

### Neutral comet assay

Cells were treated as needed and harvested using trypsin. Comet slides were prepared with the CometAssay Kit following manufacturer instructions (Trevigen 4252-040-K). Cell pellets were washed 2× with ice cold PBS and resuspended at 100K cells/ml. This suspension was mixed at a 1:10 ratio with Comet LMAgarose and 30 ul spread onto CometSlides. After agarose solidification, slides were lysed with Trevigen CometAssay Lysis Solution for 1 h followed by 30 min in 1× neutral electrophoresis buffer (0.1 M Tris, 0.3 M sodium acetate, pH 9.0) at 4°C. Slides were subject to electrophoresis for 20 min at 21 V using a Trevigen CometAssay ES II unit. DNA was precipitated for 30 min (1 M ammonium acetate in ethanol), fixed with 70% ethanol for 30 min, air-dried and stained with SYBR Gold (Invitrogen S11494) diluted in TE buffer (10 mM Tris–HCl pH 7.5, 1 mM EDTA). Images of 50–100 cells per condition were taken with a Nikon Eclipse Ti-E epifluorescence microscope equipped with a 10× objective (Plan Flour 10 × 0.30), a Photometrics CoolSnapHQ2 camera and GFP filter. Cells were analyzed using Comet Assay IV Software (Perceptive Instruments). Experiments were done in triplicate.

### Sister chromatid exchange assay

Cells were treated with 1 uM BrdU (Sigma B9285) for two cell cycles (∼48 h). At the end of the first cell cycle, 0.05 uM mitomycin C was added for 24 h. Four hours prior to harvest, colcemid (Sigma D1925) was added at 0.1 ug/ml. Cells were trypsinized, centrifuged and the pellet was resuspended in 0.075M KCl for 7 min followed by addition of 1 ml of fix (3:1 methanol/acetic acid). After centrifugation, pellets were resuspended in 10 ml of fix and incubated for 20 min at RT. Cells were washed 2× with fix and cell suspensions were dropped onto glass slides (Fisher Scientific 12-544-3) and dried in a humidified incubator at 37°C for a few min. Next day, slides were rinsed in PBS, and soaked in 0.5 ug/ml Hoescht (Sigma B1155) in PBS for 10 min. A few drops of 25 ug/ml Hoescht in PBS were dropped on slides, a coverslip was added and slides were exposed to a BLB light for 1 h. Coverslips were removed, slides immersed in SSC buffer for 15 min (0.15 M sodium chloride/0.015 M sodium citrate), rinsed with water and stained with 4% Giemsa (Ricca 3250-16) in GURR buffer (Gibco 10582-013) for 5 min. Dried slides were coverslipped using Cytoseal (Thermo Scientific 8310-4). Slides were viewed under transmitted light using a Nikon Eclipse Ti-E microscope equipped with a 40x objective (Plan Fluor, 40 × 1.30 NA, oil). For each condition, 25 normal metaphases (i.e. 2*N* ± 2 chromosomes) were analyzed for chromatid exchanges. Experiments were done in triplicate.

### siRNA transfections

For siRNA knockdown the ON-TARGET *plus* siRNA SMARTpools from Dharmacon (GE) against endogenous PDS5B (L-010362-00-0005) and SPIDR (L-025937-01-0005) were used. An ON-TARGET *plus* non-targeting pool was used as a control (D-001810-10-05). Briefly, 200 000 RPE-1, SWS1-C2 or SWSAP1-C2 cells were seeded per well of a six-well plate containing growth medium without antibiotics. Approximately 2 h later, cells were transfected with either a siRNA control, PDS5B or SPIDR. siRNAs and DharmaFECT were diluted in Opti-MEM (Gibco). A working siRNA concentration of 50 nM was used. We used 5 μl DharmaFECT transfection reagent per each six-well. Cells were split 24 h later using complete media, treated with MMS after another 48 h and finally processed for colony formation assay as described above.

### Western blots

Cells were seeded in 35 mm dishes and treated as needed. Cells were collected by trypsinization, centrifuged and washed 1x with PBS. Cells were mixed 1:1 with 2× Laemmli sample buffer (65.8 mM Tris–HCl pH 6.8, 26.3% glycerol, 2.1% SDS, 0.01% bromophenol blue, 100 mM DTT) and boiled at 99°C for 5 min. Protein extractions for immunoprecipitations are described above.

Equal amounts of protein were loaded in handcast acrylamide/bis gels and ran at 100 V using a Mini-PROTEAN Tetra Cell (Bio-Rad) in 1X running buffer (192 mM glycine, 25 mM Tris, 0.1% SDS). Protein was transferred to Immobilon-FL PVDF membranes (Millipore IPFL00010) using a Mini Trans-Blot Cell (BioRad) for 2 h at 100 V and 1× blotting buffer (192 mM glycine, 25 mM Tris, 0.01% SDS, 20% methanol). Membranes were incubated following instructions by LI-COR for near-infrared western blot detection. Briefly, following transfer membranes were blocked with Odyssey blocking buffer with TBS (LI-COR 927-50000) for 1 h at RT, and incubated with primary antibodies at 4°C overnight. After washing 4x/5 min each with TBS-T, secondary antibodies (1:20 000, LI-COR IRDye 680RD or IRDye 800CW) were incubated for 1 h at room temperature. Membranes were washed as described above and scanned using a LI-COR Odyssey CLx scanner. For scanning, laser settings were set to automatic.

### Yeast-two-hybrid and yeast-three-hybrid interactions and quantification

Yeast-two-hybrid (Y2H) and yeast-three-hybrid (Y3H) assays were performed as previously described (17) with the following modifications. For Y3Hs, pGAD, pGBD, and pRS416 vectors were co-transformed into the S. cerevisiae YPJ694a yeast strain. Yeast transformants harboring the plasmids were selected for growth on SC-LEU-TRP (Y2H) or SC-LEU-TRP-URA (Y3H) solid medium. Plates were grown for 3 days at 30°C and subsequently photographed. pGAD- and pGBD-SWS1-Val49del were made by site-directed mutagenesis (primers listed in Supplemental Table S1) and yeast-two-hybrid experiments with SWSAP1 were performed as described above.

All Y2H and Y3H images were adjusted identically for brightness and contrast using Adobe Photoshop and growth, indicating a protein interaction, quantitated using ImageJ. The value for each interrogated Y2H or Y3H interaction was normalized to the corresponding empty control vector, which was set to one. The normalized growth values are shown as a fold change relative to the negative empty control and graphed. Each experiment was performed two to three times and graphed as the average of the experiments ± standard deviation.

### Statistical analysis

GraphPad Prism (Version 7.0) was used for all statistical analyses. Data from each cell line were entered in Prism as groups and analyzed by regular two-way ANOVA with cell lines and corresponding treatment as factors. Comparisons were done by using a Tukey test with correction for multiple comparisons. Clonogenic survival data were entered in Prism in X,Y format (dose vs. relative survival) and analyzed using the dose-normalized response module. Each cell line was fitted with a non-linear regression with a least squares fit. Comparisons between fitted data were made by an extra sum-of-squares F test that compared slopes between cell lines. DNA fiber data was analyzed using a Kruskal–Wallis test followed by a Dunn's post-test for comparisons. Statistical significance was set as *P* < 0.05 and confidence intervals at 95%.

## RESULTS

### The Shu complex is important for cellular resistance to MMS and MMC

To determine the role of SWS1 and SWSAP1 in DNA repair, we used CRISPR-Cas9 to disrupt the coding sequence of the *SWS1* and *SWSAP1* genes in human RPE-1 cells (Supplementary Figure S1A). RPE-1 cells are a non-tumorigenic human retinal epithelial TERT immortalized cell line that exhibit a normal karyotype and growth parameters (27). Due to lack of SWS1 and SWSAP1 specific antibodies, we selected for knockout cell lines by genomic DNA sequencing. Two independent *SWS1* and *SWSAP1* clonal cell lines were selected for further analysis based on the presence of frameshift indels and therefore likely represent null alleles (Supplementary Figure S1B and C). One SWS1 clonal cell line contained a deletion of a single amino acid in addition to a frameshift mutation in the second allele. To confirm that this deletion altered SWS1 function, we observed a loss or reduction of SWS1 yeast-2-hybrid interaction with its binding partner SWSAP1 (Supplementary Figure S1D). Therefore, this clone is likely also deficient for SWS1 function and consistently exhibits comparable phenotypes to the other null alleles described below.

To determine whether loss of the Shu complex members *SWS1* or *SWSAP1* renders human RPE-1 cells sensitive to specific types of DNA damage, we performed clonogenic survival assays upon treatment with different DNA damaging agents. Similar to what is observed in yeast cells with Shu complex disruption (12,18–20), we find that sgSWS1 and sgSWSAP1 RPE-1 cells are primarily sensitive to the alkylating agent MMS (Figure 1A and B). MMS sensitivity was also observed in transient siRNA knockdowns in HeLa cells (15). Additionally, we find that sgSWS1 and sgSWSAP1 RPE-1 cells are sensitive to MMC (Figure 1C and D). However, the MMC sensitivity observed in sgSWS1 and sgSWSAP1 cells is modest compared with disruption of members of the Fanconi anemia pathway such as FANCD2 and is comparable to what is observed in *Swsap1^−/−^* mouse fibroblasts (28,29). Although the overall slopes are not statistically significant from one another, we find that sgSWS1 and sgSWSAP1 cell lines also exhibit a mild sensitivity to low IR doses (such as 2 and 3 Gy; Supplementary Figure S2A). In contrast, sgSWS1 and sgSWSAP1 RPE-1 cells are not significantly sensitive to cisplatin (Supplementary Figure S2B) or HU (Supplementary Figure S2C). These data suggest that the Shu complex is important to process DNA damage and its disruption confers the most sensitivity to MMS.

To better define the role of the Shu complex in tolerance of alkylation-induced DNA damage, we examined sgSWS1 and sgSWSAP1 RPE-1 cells upon exposure to MMS for DSB formation. To determine if the MMS exposure was inducing DSBs, we performed neutral comet assays in parental RPE-1 (WT), sgSWS1 and sgSWSAP1 cell lines. After one hour of MMS treatment and one hour of recovery, we observe a statistically significant and comparable increase in WT, sgSWS1, and SWSAP1 RPE-1 cell lines by neutral comet assay. More importantly, there were no differences among cell lines in the amount of DSBs induced suggesting that upon initial MMS exposure all cell lines exhibit comparable amount of DNA damage (Figure 1E and F, Supplementary Figure S3A). Since MMS is thought to result in DSB formation upon DNA replication, we then assayed these cells for S-phase arrest and observe a statistically significant and similar increase in the percentage of S phase cells in WT, sgSWS1, and sgSWSAP1 RPE-1 cells (Supplementary Figure S3B). Therefore, the increased MMS sensitivity observed above with Shu complex disruption is not due to increased DSB generation or gross changes in cell cycle dynamics upon MMS exposure.

### The Shu complex is needed for efficient RAD51 foci formation and completion of HR

**Figure 2.**
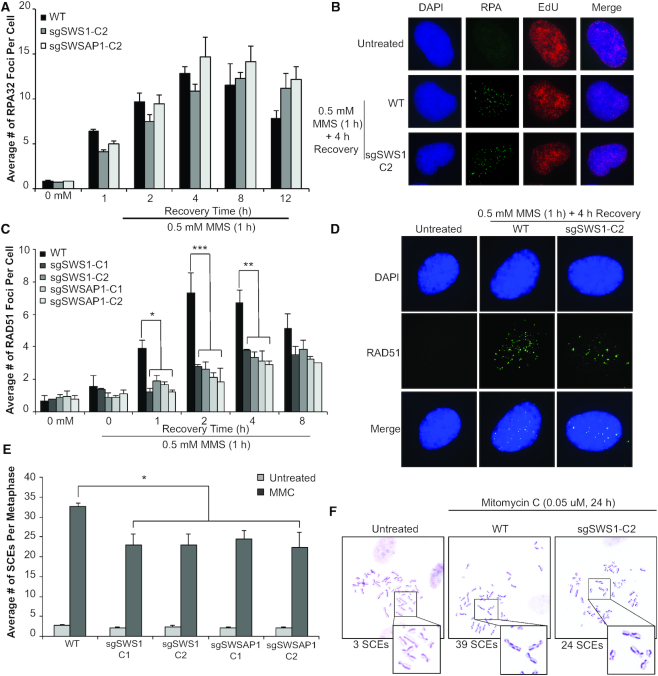
sgSWS1 and sgSWSAP1 cells exhibit reduced and delayed RAD51 foci response and fewer SCEs following DNA damage. (**A**) The average number of RPA32 foci per cell were measured in EdU-positive cells by fluorescent microscopy after MMS treatment (0.5 mM for 1 h) in WT, sgSWS1-C2 and sgSWSAP1-C2 RPE-1 cells. Three independent experiments were performed (50 cells per experiment). The standard error of the mean was graphed, and statistical differences calculated using two-way ANOVA (cell line and treatment as factors) followed by a Tukey test for multiple comparisons. (**B**) Representative images of A) showing DAPI (blue), RPA32 foci (green; RPA) and EdU (red) staining in untreated RPE-1 and MMS-treated WT and sgSWS1-C2 RPE-1 cells. (**C**) The average number of RAD51 foci per cell were measured by fluorescent microscopy after MMS treatment (0.5 mM for 1 h) in WT, sgSWS1-C1, sgSWS1-C2, sgSWSAP1-C1, and sgSWSAP1-C2 cells. Three independent experiments were performed (50 cells per experiment). The standard error of the mean was graphed and statistical differences calculated using two-way ANOVA (*P< 0.05, **P< 0.005, ***P< 0.0005) (cell line and treatment as factors) followed by a Tukey test for multiple comparisons. (**D**) Representative images of C) showing DAPI (blue) and RAD51 foci (green) in untreated RPE1 and MMS-treated WT and sgSWS1-C2 RPE-1 cells. (**E**) The average number of SCEs per metaphase were calculated from WT, sgSWS1-C1, sgSWS1-C2, sgSWSAP1-C1, and sgSWASP1-C2 RPE-1 cells after 24 h treatment with 0.05 uM mitomycin C (MMC). Three independent experiments were performed (25 normal [2N±2] metaphases were scored per experiment). Standard error of the mean was graphed and statistical differences calculated using a two-way ANOVA with cell line and treatments as factors (*P<0.05). ANOVA was followed by a Tukey test for multiple comparisons. (**F**) Representative images of (**E**) showing SCEs in untreated, WT and sgSWS1-C2 MMC-treated cells. The box indicates a zoomed-in section of the metaphase spread, and the number of SCEs observed is indicated below.

Next, we asked if Shu complex disruption would lead to altered HR dynamics. To test this, we assayed sgSWS1 and sgSWSAP1 cells for changes in RPA and RAD51 foci induction and sister chromatid exchanges. During HR, after DSB formation, the DNA ends are resected and RPA-coated (1). RPA recruitment to ssDNA can be observed by monitoring cells for RPA32 foci using immunofluorescence. At the same time, replicating cells were co-stained using EdU and RPA32 foci were only observed in EdU-positive cells highlighting that repair of MMS-induced DNA damage is in S phase. We determined the average number of RPA32 foci in replicating cells in WT, sgSWS1, and sgSWSAP1 RPE-1 cells following 1 h of MMS exposure for up to 12 h of recovery (Figure 2A and B). In WT cells, we observe an increase in RPA32 foci one hour after MMS exposure which further increases at four hours and is significantly reduced after 12 hours of recovery (Figure 2A; *P* = 0.0286). Although sgSWS1 and sgSWSAP1 exhibit similar RPA32 foci induction at 4 hours of recovery, we do not observe a significant reduction in RPA32 foci comparing the 4 and 12 h time points (Figure 2A; sgSWS1 *P*>0.999 and sgSWSAP1 *P* = 0.554). The induction of RPA foci over time suggests that DNA end resection is likely occurring in the absence of the Shu complex. However, it is possible that Shu complex disruption may result in slower or delayed RPA removal, which could be indicative of RAD51 loading defects, the subsequent HR step. Therefore, we analyzed the average number of RAD51 foci in WT, sgSWS1, and sgSWSAP1 cells following MMS exposure and observe a significant reduction and delay in RAD51 foci after 1, 2 and 4 h of recovery (Figure 2C and D). In addition, sgSWS1 and sgSWSAP1 cells also exhibit overall fewer cells with RAD51 foci after one and two hours of recovery (Supplementary Figure S4). Therefore, SWS1 and SWSAP1 are important for efficient RAD51 recruitment into DNA repair foci following MMS exposure during S phase.

Since we observe a reduced and delayed RAD51 foci response in Shu complex mutant knockout cells, we wanted to address if Shu complex disruption would result in fewer recombination events. In mouse embryonic fibroblasts disruption of the RAD51 paralog, *RAD51C*, results in decreased sister chromatid exchanges (SCEs, which are HR-mediated crossovers) following MMC exposure (9). Since human sgSWS1 and sgSWSAP1 RPE-1 cells are MMC sensitive, we asked whether these cells would exhibit fewer SCEs following MMC exposure. Consistent with a role in promoting RAD51-mediated HR, we observe a 30% reduction in the average number of SCEs in sgSWS1 and sgSWSAP1 cells (Figure 2E and F). Together, these results support a model where the human Shu complex promotes RAD51-mediated recombination.

### The Shu complex is needed to restart, but not protect, stalled replication forks

**Figure 3.**
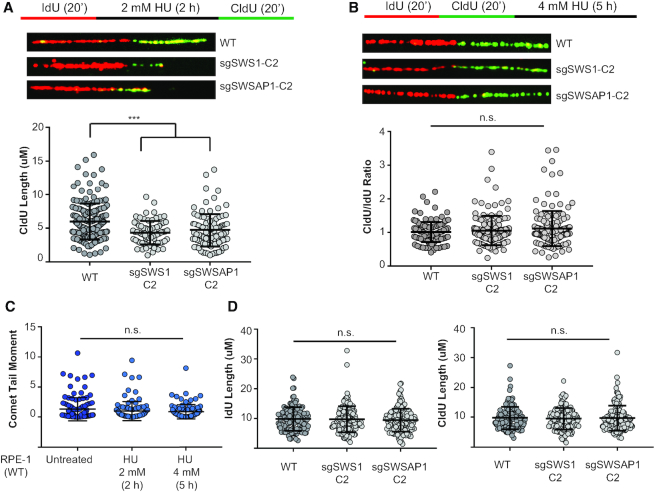
The Shu complex is needed to restart, but not protect, stalled replication forks. (**A**) Fork restart schematic of DNA labelling is shown where the cells were pulsed for 20 min with IdU, the forks were then stalled for 2 h with 2 mM HU, followed by a 20 min CldU pulse to analyse fork restart. A representative image for each genotype is shown and the CldU length (μM) quantitated for WT, sgSWS1-C2, and sgSWSAP1-C2 cells. A scatter plot of all acquired data points is shown (WT *N* = 186, sgSWS1-C2 *N* = 73, sgSWSAP1-C2 *N* = 101). Two independent experiments were performed and with 73–186 total fibers analyzed. Error bars represent the mean and standard error of the mean for each data set. Statistical significance was calculated using a Kruskal-Wallis test. ***P< 0.0005. (**B**) Fork protection schematic of DNA labelling is shown where the cells were pulsed with IdU for 20 min followed by CldU for 20 min and treated with 4 mM HU for 5 h. A representative image for each genotype is shown and the CldU/IdU tract length ratios were quantitated for WT, sgSWS1-C2, and sgSWSAP1-C2 cells. Two independent experiments were performed and 130–151 total fibers analysed. A scatter plot of all acquired data points is shown (WT N = 130, sgSWS1-C2 *N* = 143, sgSWSAP1-C2 *N* = 151). Error bars represent the mean and standard error of the mean for each data set. Statistical significance was calculated using a Kruskal-Wallis test (n.s. indicates not significant). (**C**) DSBs were measured by determining the neutral comet tail moment in untreated RPE-1 cells and after exposure to 2 mM HU for 2 and 5 h. Experiments were performed in duplicate with 50 comets analyzed per experiment. Data are expressed as mean comet tail moment with standard error of the mean graphed. Statistical significance was calculated using a one-way ANOVA. (**D**) IdU and CldU fiber length after pulsing with IdU for 20 min followed by CldU for 20 min. Two independent experiments were performed. A scatter plot of all acquired data points is shown (WT N = 130, sgSWS1-C2 *N* = 143, sgSWSAP1-C2 *N* = 151). Error bars represent the mean and standard error of the mean for each data set. Statistical significance was calculated using a Kruskal–Wallis test (n.s. indicates not significant).

Since RAD51 loading in response to MMS was delayed in Shu knockout cells, we asked whether the Shu complex also functions in replication fork restart and protection, processes known to be RAD51 dependent (30–36). Similarly, proteins that regulate RAD51, including the canonical RAD51 paralogs, are also implicated in these processes upon fork stalling by HU (32,33,37,38). To address if the Shu complex functions in replication fork restart and protection, we performed DNA fiber analysis. To examine fork restart, we first pulsed the replicating DNA with IdU and then stalled the forks with HU for 2 h and subsequently pulsed the DNA with CldU (Figure 3A). By measuring the CldU track length following the IdU labelled DNA, we observe a statistically significant decrease in restarted forks in sgSWS1 and sgSWSAP1 cells compared to WT (Figure 3A). These results suggest that the Shu complex functions during replication fork restart.

Next, we determined if SWS1 and SWSAP1 would have a role in replication fork protection. To do this, we consecutively pulsed the cells first with IdU and then with CldU, and then subsequently treated the cells with HU (Figure 3B). Unlike fork restart, we do not observe decreases in replication track lengths by measuring the CldU to IdU ratio in sgSWS1 and sgSWSAP1 cells (Figure 3B). Therefore, the Shu complex does not exhibit a replication fork protection function. These results suggest that replication fork protection and restart can be uncoupled.

Since we observe defects in replication fork restart in Shu complex-disrupted cells, we wanted to determine if DSBs are forming under the HU conditions utilized during the replication fork protection assays. To do this, we performed neutral comet assays in RPE-1 WT cells and do not observe DSB induction (Figure 3C). Additionally, suggesting that Shu knockout cells do not have grossly altered replication dynamics compared to WT cells, the length of the DNA fibers from the IdU and CldU pulses were similar to each other and between cell lines (Figure 3D). Therefore, the fork restart function of the Shu complex occurs independently of DSB formation.

### BioID identifies novel Shu complex interacting proteins, SPIDR and PDS5B

**Figure 4.**
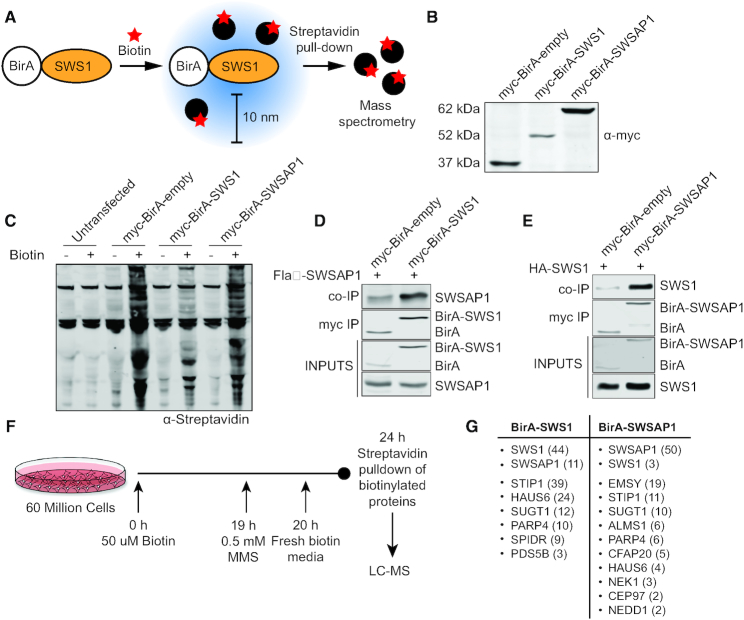
BioID analysis to identify novel Shu complex interacting proteins. (**A**) Schematic of BirA-tagged SWS1, which upon biotin exposure (star) will biotinylate proteins within a 10 nanometer (nm) range. Biotinylated proteins are pulled-down with streptavidin-coated magnetic beads and elutes are analyzed by mass spectrometry. (**B**) Whole cell lysates from RPE-1 cells stably expressing myc-BirA, myc-BirA-SWS1 and myc-BirA-SWSAP1 fusion proteins were western blotted using an anti-myc antibody. (**C**) Untreated and biotin treated (50 uM, 24 h) whole cell lysates from parental RPE-1 (untransfected), myc-BirA-empty, myc-BirA-SWS1, myc-BirA-SWSAP1 RPE-1 cell lines were western blotted for biotinylated proteins using an anti-streptavidin antibody. (**D**) myc-BirA-empty and myc-BirA-SWS1 RPE-1 cells were transiently transfected with Flag-SWSAP1. Myc-BirA was immunoprecipitated using myc-conjugated beads (myc IP) and western blotted for Flag-SWSAP1 (co-IP) using an anti-Flag antibody. Inputs represent 10% of the protein lysate. (**E**) myc-BirA-empty and myc-BirA-SWSAP1 RPE-1 cells were transiently transfected with HA-SWS1. Myc-BirA was immunoprecipitated using myc-conjugated beads (myc IP) and western blotted for HA-SWS1 (co-IP) using an anti-HA antibody. Inputs represent 10% of the protein lysate. (**F**) Treatment schematic for BioID experiments. Sixty million cells per cell line (myc-BirA-empty, myc-BirA-SWS1 and myc-BirA-SWSAP1) were grown and treated with 50 uM biotin for 24 h. During the biotin treatment, 0.5 mM MMS was added to the media for 1 h and the cells recovered for 4 h. Whole cell lysates were subject to streptavidin pulldown and biotinylated proteins were identified by liquid chromatography–mass spectrometry–mass spectrometry (LC–MS–MS). Two independent experiments were performed. (**G**) Table of enriched genome stability proteins detected by LC–MS–MS in myc-BirA-SWS1 and myc-BirA-SWSAP1 cell lines. Peptide counts for each protein from one experiment are in parenthesis. A complete list of proteins identified is found in the Supplemental Table S2.

The budding yeast Shu complex is a heterotetramer, and therefore, we wondered whether there may be additional Shu complex members in human cells. To identify potential human Shu complex interacting proteins, we used the BioID technique (39) to screen for novel SWS1 and SWSAP1-interacting proteins after MMS-induced DNA damage. To do this we created stable RPE-1 cell lines that express SWS1 or SWSAP1 attached to the bacterial biotin ligase BirA harboring a mutation that makes it promiscuously biotinylate proteins in close proximity upon biotin addition (10 nm; Figure 4A and B). As a control, we used a non-conjugated stable cell line expressing BirA (Figure 4B). The BirA construct also contains a myc tag. Using these cell lines, we observe an increase in biotinylated proteins upon biotin addition in the myc-BirA-SWS1 and myc-BirA-SWSAP1 cell lines but not in cells lacking the myc-BirA construct (compare untransfected plus biotin addition with myc-BirA constructs; Figure 4C). To determine if the myc-BirA tag may be interfering with either SWS1 or SWSAP1 known protein interactions, we performed co-immunoprecipitation (co-IP) experiments with the myc-BirA-SWS1 and myc-BirA-SWSAP1 cells with transiently transfected Flag-SWSAP1 or HA-SWS1, respectively (Figure 4D and E). Importantly, both myc-BirA-SWS1 and myc-BirA-SWSAP1 maintain their protein interactions with their binding partners. To perform BioID, we treated cells with biotin, added MMS for one hour, and allowed the cells to recover for four hours, which is when we observe a significant increase in RAD51 foci (Figures 4F and 2C). We then pulled down the biotinylated proteins using streptavidin beads and analyzed the elutes by mass spectrometry. Commonly found contaminants were eliminated using the CRAPome database (40). Proteins were only considered to potentially interact with SWS1 or SWSAP1 if they were not observed in the myc-BirA-empty cell line or they were enriched three-fold and at least two unique peptides were recovered (Supplemental Table S2; Figure 4G).

**Figure 5.**
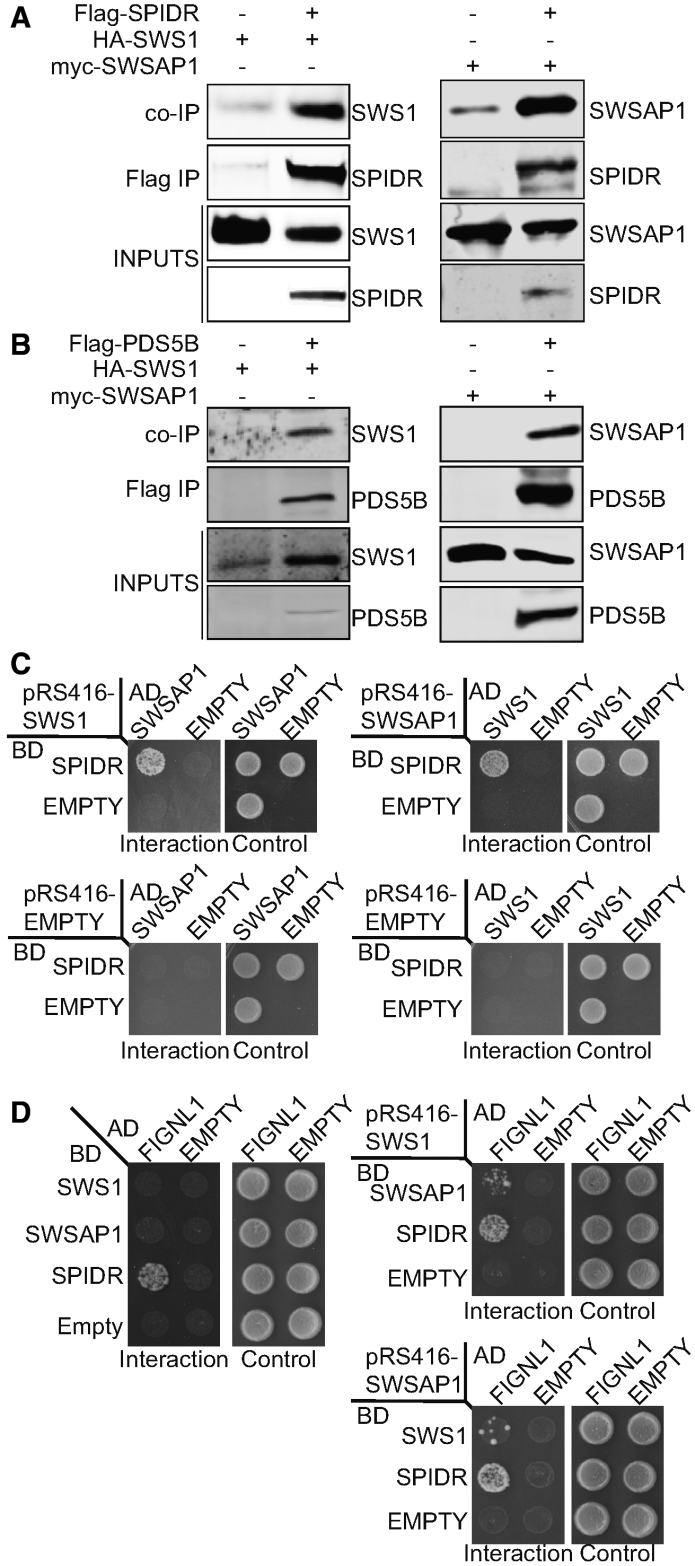
BioID identifies SPIDR and PDS5B as novel binding partners of the Shu complex. (**A**) RPE-1 cells were transiently transfected with either HA-SWS1 or myc-SWSAP1 alone or with Flag-SPIDR. Flag-SPIDR was immunoprecipitated using anti-Flag-conjugated beads (Flag IP) and western blotted for SWS1 or SWSAP1 (co-IP) using anti-HA or anti-myc, antibodies, respectively. Inputs represent 10% of the protein lysate. (**B**) RPE-1 cells were transiently transfected with either HA-SWS1 or myc-SWSAP1 alone or with Flag-PDS5B N-terminal fragment (1–529 amino acids). Flag-PDS5B was immunoprecipitated using anti-Flag-conjugated beads (Flag IP) and western blotted for SWS1 or SWSAP1 (co-IP) using anti-HA or anti-myc antibodies, respectively. Inputs represent 10% of the protein lysate. (**C**) The PJ694a yeast strain was transformed with three plasmids; 1) a plasmid where SPIDR was fused to the GAL4-DNA binding domain (BD; pGBD-SPIDR), 2) a plasmid where either SWSAP1 or SWS1 was fused to the GAL4-DNA activating domain (AD; pGAD-SWSAP1 or pGAD-SWS1), and 3) a plasmid that constitutively expressed either SWS1 or SWSAP1 (pRS416-SWS1, pRS416-SWSAP1). A yeast-three-hybrid interaction between SPIDR and SWSAP1 or SWS1 was assayed by platting the yeast on SC-LEU-TRP-URA-HIS (Interaction, indicated by growth) and compared to the loading control SC-LEU-TRP-URA (Control). Empty BD (pGBD), AD (pGAD), and pRS416 plasmids were used as negative controls. (**D**) The PJ69a yeast strain was transformed with a plasmid where FIGNL1 was fused to the GAL4-DNA activating domain (AD; pGAD-FIGNL1) and 2) a plasmid where SWSAP1, SWS1, or SPIDR was fused to the GAL4-DNA binding domain (BD; pGBD-SWSAP1, pGBD-SWS1, pGBD-SPIDR). For the yeast-three-hybrids, a third plasmid that constitutively expressed either SWS1 or SWSAP1 (pRS416-SWS1, pRS416-SWSAP1) was also co-transformed. A yeast-two-hybrid interaction between FIGNL1 with SWS1, SWSAP1, or SPIDR was assayed by plating the yeast on SC-LEU-TRP-HIS (Interaction, indicated by growth) and compared to the loading control SC-LEU-TRP (Control). A yeast-three-hybrid interaction between SPIDR and SWSAP1 or SWS1 was assayed by plating the yeast on SC-LEU-TRP-URA-HIS (Interaction, indicated by growth) and compared to the loading control SC-LEU-TRP-URA (Control). Empty BD (pGBD), AD (pGAD), and pRS416 plasmids were used as negative controls.

Using this BioID approach, we sought to identify novel Shu complex members. We validated that SWS1 and SWSAP1 are in close proximity to each other as well as to additional proteins that function to promote genome stability including DNA repair, kinetochore, and mitotic spindle formation (Figure 4G). In particular, SPIDR, PDS5B, and EMSY are already known to function during HR and their knockdown, similar to the Shu complex, results in decreased RAD51 foci formation following DNA damage (25,41–44). To determine if SWS1 or SWSAP1 interact with SPIDR, PDS5B, or EMSY, we performed co-IP experiments with HA-tagged SWS1 or myc-tagged SWSAP1 and Flag-tagged SPIDR, PDS5B, or untagged EMSY. Note that we were only able to express a 1–529 amino acid N-terminal fragment of PDS5B in RPE-1 cells. We used benzonase to rule out DNA-mediated protein-protein interactions. Both FLAG-SPIDR and FLAG-PDS5B co-IP HA-SWS1 and MYC-SWSAP1 (Figure 5A and B). These protein–protein interactions are also observed when FLAG-SPIDR or FLAG-PDS5B were co-immunoprecipitated (Supplementary Figure S5A and S5B; note that only MYC-SWSAP1 reliably co-IPs FLAG-PDS5B). Furthermore, suggesting that SPIDR’s interaction with SWS1 and SWSAP1 is likely direct, we observe a yeast-three-hybrid interaction between SPIDR with SWS1 and SWSAP1 (Figure 5C and Supplementary Figure S5C). By yeast-three-hybrid, we find that SPIDR’s N terminus (1–515 amino acids) likely mediates these protein-protein interactions and this is the same region of SPIDR that interacts with RAD51 (Supplementary Figure S5D) (43). In contrast to SPIDR and PDS5B, EMSY does not co-IP with SWS1 or SWSAP1 even upon MMS exposure (Supplementary Figure S6). Therefore, EMSY is not a bonafide binding partner of SWS1 and SWSAP1 and is most likely in close proximity to the Shu complex during DNA repair. Finally, a previous study identified FIGNL1 to directly interact with SPIDR (45) and a recent study indicates an interaction with SWSAP1 (29). Although we did not identify FIGNL1 in our BioID mass spectrometry analysis of SWS1 or SWSAP1 interacting proteins, we sought to determine if FIGNL1 would exhibit a yeast-two-hybrid or a yeast-three-hybrid interaction with either SWS1 or SWSAP1. In contrast to SPIDR, we observe no yeast-two-hybrid interaction or an extremely weak yeast-three-hybrid interaction between FIGNL1 with either SWS1 or SWSAP1 (Figure 5D and Supplementary Figure S5E–G). Together these experiments reveal SPIDR and PDS5B to be novel SWS1 and SWSAP1 interacting partners.

**Figure 6.**
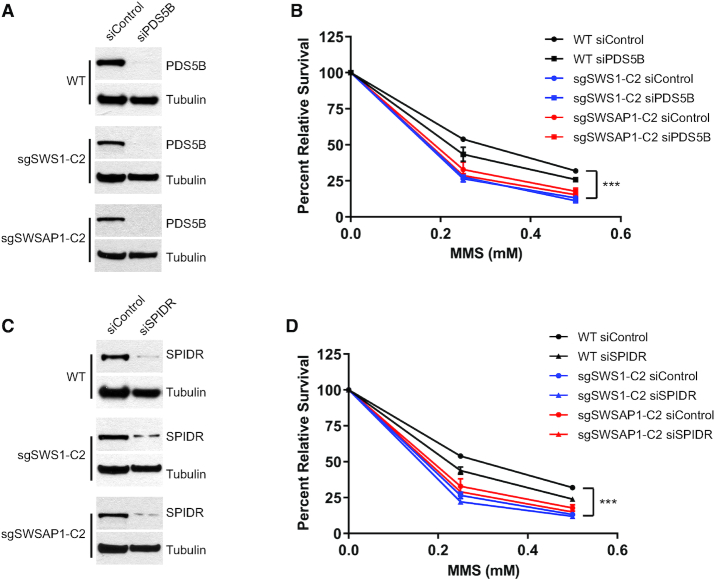
SWS1-SWSAP1 function in the same pathway as SPIDR and PDS5B. (**A**) Western blots of PDS5B protein levels in WT RPE-1, sgSWS1-C2 and sgSWSAP1-C2 cell lines after PDS5B siRNA knockdown. Tubulin was used as a loading control. (**B**) Clonogenic survival assays of WT parental RPE-1, sgSWS1-C2, and sgSWSAP1-C2 cell lines following PDS5B knockdown using siRNA and MMS exposure. PDS5B was knocked down using siRNA for 72 hours before 1 hour treatment with MMS at the indicated concentrations. Treated cells were seeded at colony forming density and stained with crystal violet 8 days post-seeding. Mean percent survival relative to untreated cells and standard error of the mean are shown. All experiments were performed in duplicate. Statistical differences were calculated by fitting a non-linear regression curve to each data set and comparing slopes between WT and sgSWS1 cells. (**C**) Western blots of SPIDR protein levels in WT RPE-1, sgSWS1-C2 and sgSWSAP1-C2 cell lines after SPIDR siRNA knockdown. Tubulin was used as a loading control. (**D**) Same as (A) except that SPIDR was knocked down using siRNA.

To investigate if SPIDR and PDS5B genetically function in the same pathway as SWS1 or SWSAP1 with respect for MMS resistance, we used siRNA to knock down PDS5B or SPIDR in WT parental RPE-1, sgSWS1 and sgSWSAP1 cell lines (Figure 6A and C). Consistent with a function for PDS5B or SPIDR in HR (42,43), siRNA knock down of either PDS5B or SPIDR results in MMS sensitivity compared to the WT parental RPE-1 cells (Figure 6B; *P* = 0.0025 siPDS5B or Figure 6D; *P* = 0.0008 siSPIDR compared to RPE-1). Although siPDS5B or siSPIDR cells are MMS sensitive, their MMS sensitivity was less than sgSWS1 and sgSWSAP1 RPE-1 cells (Figure 6B; *P* < 0.0001 siPDS5B or Figure 6D; *P* < 0.0001 siSPIDR compared to either sgSWS1 or sgSWSAP1). Suggesting that PDS5B and SPIDR function in the same pathway as SWS1 and SWSAP1, our genetic data indicates that sgSWS1-siPDS5B or sgSWSAP1-siPDS5B double mutants exhibit the same MMS sensitivity as an sgSWS1 or sgSWSAP1 single mutant (Figure 6B; *P* = 0.9345 for SWS1; *P* = 0.1672 for SWSAP1). The same is observed for sgSWS1-siSPIDR and sgSWSAP1-siSPIDR double mutants (Figure 6D; *P* = 0.190 for SWS1; p-0.1155 for SWASP1). Therefore, our results indicate that PDS5B and SPIDR genetically function in the same pathway as SWS1 and SWSAP1.

## DISCUSSION

Here we show that the human Shu complex is required for efficient replication-associated HR following MMS exposure. By creating CRISPR/Cas9 SWS1 and SWSAP1 knockouts in human RPE-1 cell, we were able to uncover new functions for the human Shu complex members in a non-cancerous cell line. We demonstrate that loss of the Shu complex members, *SWS1* or *SWSAP1*, result in MMS and MMC sensitivity, a delayed and reduced RAD51 foci response, fewer sister chromatid exchanges, and defects in replication fork restart. Furthermore, we identified SPIDR and PDS5B as novel Shu complex interacting partners. Together our results support a model where the human Shu complex is important for tolerance of alkylation-induced DNA damage during replication by promoting RAD51-mediated functions.

One unique aspect of the human Shu complex is its primary sensitivity to the alkylating agent MMS, a feature that is also shared in other eukaryotes including yeast and worms (3,12,17–19,21). MMS-induced DNA damage is typically repaired using the base excision repair (BER) pathway (46,47). However, during DNA replication, collision of the replication fork with a BER intermediate can result in replication fork stalling and/or collapse. It is in this context that the HR machinery would be important for error-free damage tolerance. Consistent with a function for the Shu complex in processing of alkylation-induced DNA damage intermediates, Shu complex-disrupted yeast cells exhibit increased MMS sensitivity when combined with BER mutants such as the DNA glycolyase *MAG1*, which removes the N3-methyl-adenine leaving an apurinic/apyrimidinic (AP) site, and the AP endonucleases *APN1* and *APN2* which convert the AP site into a nick (21,48). Based upon our studies here, we propose that the human Shu complex becomes important for tolerance of MMS-induced DNA damage intermediates that arises specifically during DNA replication. Consistent with this model, MMS-exposed Shu knockout cells exhibit delayed resolution of RPA foci and a delayed and decreased RAD51 response in our sgSWS1 and sgSWSAP1 cells specifically in replicating cells as measured by EdU staining. Finally, Shu complex disruption results in a defect in replication restart upon fork stalling by HU. These results are unique from what is observed by disrupting the canonical RAD51 paralogs, such as RAD51C and XRCC3, where both replication fork restart and protection were inhibited (37). Since RAD51 also mediates fork restart after HU (31), the fork restart defect observed in the Shu complex knockout cells could be a result of impaired or delayed RAD51 loading at stalled forks. In this context, RAD51 has been proposed to mediate fork reversal allowing the formation of a Holliday junction intermediate (i.e. chicken foot) whose DNA end may facilitate recombination-dependent fork restart (31,35). It is also possible that the Shu complex functions in stabilization or formation of RAD51 filaments on the gap in the replication fork either before replication fork regression or after its restoration. Our findings here cannot distinguish between these different possibilities. Together, these results suggest that the human Shu complex function is likely replication dependent and may be distinct from the other RAD51 paralogs.

Within the context of DNA replication, the budding yeast Shu complex promotes Rad51 presynaptic filament assembly. Our results suggest that the human Shu complex may perform an analogous function since SWS1 or SWSAP1 disruption decreases RAD51 foci formation and results in fewer SCEs. These results are consistent with biochemical studies from yeast showing a 2–3-fold stimulation of Rad51 filament formation in the presence of the yeast Shu complex in combination with the other yeast Rad51 mediators [Rad55-Rad57 and Rad52; (13)]. In agreement with this notion, a decrease in RAD51 foci after radiation exposure and MMS were observed in transformed human cancer cells upon siRNA knockdown (14,15). Similarly, SWS1 and SWSAP1 knockout mice have recently demonstrated that the mouse Shu complex promotes assembly of RAD51 and DMC1 on early meiotic HR substrates and that this is crucial for crossover homeostasis and proper oogenesis and spermatogenesis (49). Like the other RAD51 paralog containing complexes, these data point to a role for the Shu complex as a RAD51 mediator (2,3).

In other species where the Shu complex has been studied, it is a multimeric complex composed of RAD51 paralogs and a SWIM domain-containing Shu2/SWS1 protein family member (14,16). Since the yeast complex is a heterotetramer, it remained unknown if the human Shu complex contained additional members. To reveal new binding partners of the Shu complex, we used a BioID approach (39) and identified PDS5B and SPIDR as novel Shu complex interacting partners. Both PDS5B and SPIDR function during HR and regulate RAD51 foci formation (42,43). SPIDR is a proposed scaffolding protein that binds to RAD51 in its N-terminus (43), which is the same region where we observe its interaction with SWS1 and SWSAP1. The cohesion-associated protein PDS5B directly interacts with BRCA2 and functions primarily during S phase to facilitate HR following aphidicolin or HU-induced fork collapse (41). Additionally, PDS5B is enriched at HU-stalled forks (Sirbu *et al.* 2013). It is worth highlighting that BRCA2 was enriched in one of our BioID experiments, but this interaction was not confirmed. However, consistent with a role for the Shu complex as a RAD51 mediator, the presence of these bonafide BRCA2 interactors (PDS5B and EMSY) in our BioID experiments suggest that the Shu complex may be acting in close spatial and temporal proximity to BRCA2. Although we did not pull down FIGNL1 in our BioID screen for SWS1 or SWSAP1 interacting proteins, it is interesting to note that a recent study identified SWSAP1 to interact with FIGNL1 and this interaction protects RAD51 filament formation from FIGNL1 anti-recombinase activity (29). It is possible that this might represent a conserved mechanism as we showed that the yeast Shu complex prevents Srs2 recruitment to DSB sites (50). It is possible that in human cells this interaction may be SPIDR-mediated as SPIDR also binds to FIGNL1 (45). A similar antagonistic mechanism has been reported between *Arabidopsis thaliana* FIGL1 and BRCA2 (51).

Deficiency in the canonical RAD51 paralogs, such as RAD51C and RAD51D, are linked to familial breast and ovarian cancer predisposition (2). As for the Shu complex, a homozygous mutation in the SWS1 gene (also known as ZSWIM7) has been associated with colorectal adenomatous polyposis (52). Additionally, recent GWAS studies has proposed SWS1 as a susceptibility gene for chronic obstructive pulmonary disease (COPD) and cardiovascular disease (53,54). These epidemiology studies highlight the importance of HR factors in the etiology of cancer and other diseases. Here, we examined the function of Shu complex members SWS1 and SWSAP1 during HR to elucidate the biology of these elusive, yet highly conserved and relevant, HR mediators.

## Supplementary Material

gkz738_Supplemental_Files

## Notes

Present address: Julieta Martino, Cell Cycle and Genomic Stability Laboratory, Fundación Instituto Leloir, Av. Patricias Argentinas 425, C1405BW, Buenos Aires, Argentina.

Present address: Tatiana N. Moiseeva, Department of Chemistry and Biotechnology, Akadeemia tee 15, Tallinn University of Technology, Tallinn, Estonia.
