# Supplementary material for: The human Shu complex functions with PDS5B and SPIDR to promote homologous recombination
Source: Nucleic Acids Res. 2019 Sep 6;47(19):10151–65. doi: 10.1093/nar/gkz738 (PMC6821187; doi:10.1093/nar/gkz738)
Supplement: gkz738_Supplemental_Files [file gkz738_supplemental_files.zip › Second Revision Human Shu paper-Supp_Submitted2-.docx]

**Supplemental Table 1: List of plasmids and primers used in this study.**

| **Lab Stock Name** | **Plasmid** | **Insert** | **Primers** | **Restriction sites and markers** |
| --- | --- | --- | --- | --- |
| pKB512 | pcDNA3.1 mycBioID | SWS1 | GCGGCGGCCGCGGATGGCCGTAGTGTTGCC (FW)  GCGGGATCCTTATGCTTCTTGTTTCTTCTCCATC (RV) | NotI  BamHI  *AMP^R^* |
| pKB514 | pcDNA3.1 mycBioID | SWSAP1 | GCGGCGGCCGCGGATGCCTGCCGCCGGACC (FW)  GCGGGATCCTCAGGGCTGGCCTCCAGAG (RV) | NotI  BamHI  *AMP^R^* |
| pKB51 | pGAD | SWS1 | GCGGAATTCATGGCCGTAGTGTTGCCGGCGGTTG (FW)  GCGGTCGACTTATGCTTCTTGTTTCTTCTCCATC (RV) | MfeI  SalI  Leu, *AMP^R^* |
| pKB78 | pGBD | SWS1 | GCGGAATTCATGGCCGTAGTGTTGCCGGCGGTTG (FW)  GCGGTCGACTTATGCTTCTTGTTTCTTCTCCATC (RV) | MfeI  SalI  Trp, *AMP^R^* |
| pKB906 | pGAD | SWS1-Val49del | GGCCTTGGACCTAGATCGACAGTCCATC (FW)  GATGGACTGTCGATCTAGGTCCAAGGCC (RV) | Leu, *AMP^R^* |
| pKB907 | pGBD | SWS1-Val49del | GGCCTTGGACCTAGATCGACAGTCCATC (FW)  GATGGACTGTCGATCTAGGTCCAAGGCC (RV) | Trp, *AMP^R^* |
| pKB52 | pGAD | SWSAP1 | GCGGAATTCATGCCTGCCGCCGGACCGCCTTTG (FW)  GCGGTCGACTCAGGGCTGGCCTCCAGAGCTTGAAC (RV) | EcoRI  SalI  Leu, *AMP^R^* |
| pKB60 | pGBD | SWSAP1 | GCGGAATTCATGCCTGCCGCCGGACCGCCTTTG (FW)  GCGGTCGACTCAGGGCTGGCCTCCAGAGCTTGAAC (RV) | EcoRI  SalI  Trp, *AMP^R^* |
| pKB898 | pGAD | FIGNL1 | CTATCGAATTCATGCAGACCTCCAGCTCTAG (FW)   \| GATAGGTCGACTTACTTTCCACAACCAAAAG (RV) \| \| --- \| | EcoRI  SalI  Leu, *AMP^R^* |
| pKB632 | pGAD | SPIDR | gcgGGATCCatgccccgcggcag (FW)  gcgGTCGACctagtgttctgcagaggccc (RV) | BamHI  SalI  Leu, *AMP^R^* |
| pKB633 | pGBD | SPIDR | gcgGGATCCatgccccgcggcag (FW)  gcgGTCGACctagtgttctgcagaggccc (RV) | BamHI  SalI  Trp, *AMP^R^* |
| pKB544 | pCMV3-C-FLAG | SPIDR | NA | From Sino Biological  accession# NM_001080394.3  *KAN^R^* |
| pKB545 | pCMV3-C-FLAG | PDS5B | NA | From Sino Biological  accession# BC039256  *KAN^R^* |
| pKB758 | pGBD | SPIDR 1-515 aa | CCGGAATTCATGCCCCGCGGCAG (FW)  CGCGGATCCCTCGAGTTCCAGCTGGGTCTG (RV) | EcoRI  BamHI  Trp, *AMP^R^* |

**Supplemental Figure 1**

**
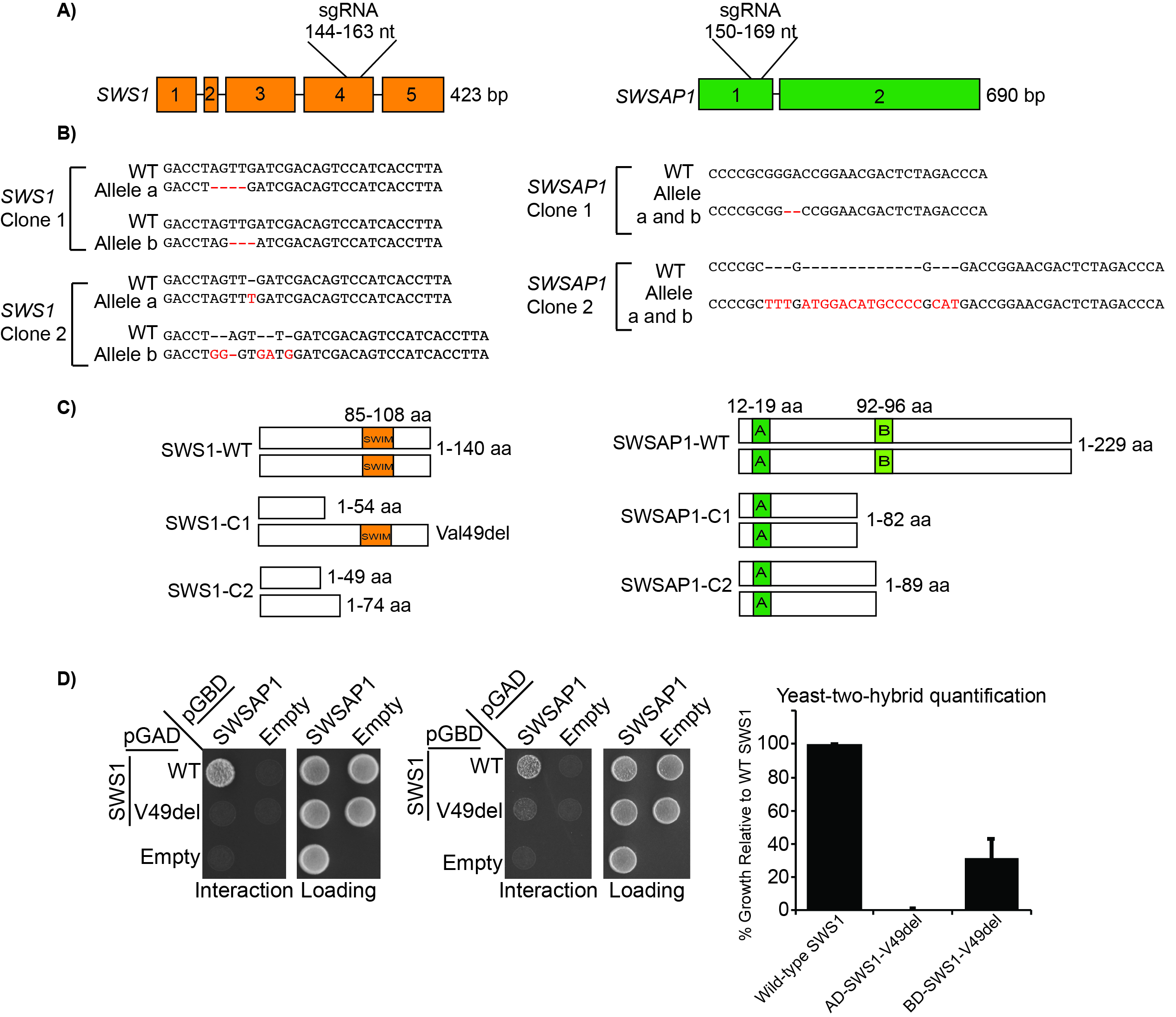
**

**Figure S1. CRISPR-Cas9 was used to create *sgSWS1* and *sgSWSAP1* RPE-1 cell lines. A)** Schematic of gRNA target sites in *SWS1* (144-163 nt) and *SWSAP1* (150-163 nt) genes. The *SWS1* (also known as *ZSWIM7*) and *SWSAP1* genes are depicted where the exons are indicated with orange boxes (*SWS1*) or green boxes (*SWSAP1*). **B)** Comparison of wild-type *SWS1* and *SWSAP1* genes in the parental RPE-1 cells versus CRISPR-edited sequences in sgSWS1 clones 1 and 2 for each allele (a and b) and sgSWSAP1 clones 1 and 2 for each allele (a and b). Deleted nucleotides (shown as dashes) and any base pair insertions are indicated in red. **C)** Schematic of predicted protein products based on sequenced genome edits in B). SWS1-WT and SWSAP1-WT proteins with conserved SWIM domain (amino acids 85-108; orange) and Walker A (A; amino acids 12-19; green) or Walker B (B; amino acids 92-96; light green) indicated. **D)** Yeast-two-hybrid analysis shows that SWS1-V49del results in reduced interaction with SWSAP1. The PJ694a yeast strain was transformed with a plasmid where SWS1 or SWS1-V49del was fused to the GAL4-DNA activating domain (AD; pGAD-SWS1 or pGAD-SWS1-V49del), or a plasmid where either SWSAP1 was fused to the GAL4-DNA binding domain (BD; pGBD-SWSAP1). In addition, the same proteins were also probed for interaction in the reciprocal vectors. A yeast-two-hybrid interaction between SWS1 and SWSAP1 was assayed by plating the yeast on SC-LEU-TRP-HIS (Interaction, indicated by growth) and compared to the loading control SC-LEU-TRP (Loading). Quantification of growth relative to wild type is shown as the mean of two experiments plotted with standard error of the mean.

**Supplemental Figure 2**


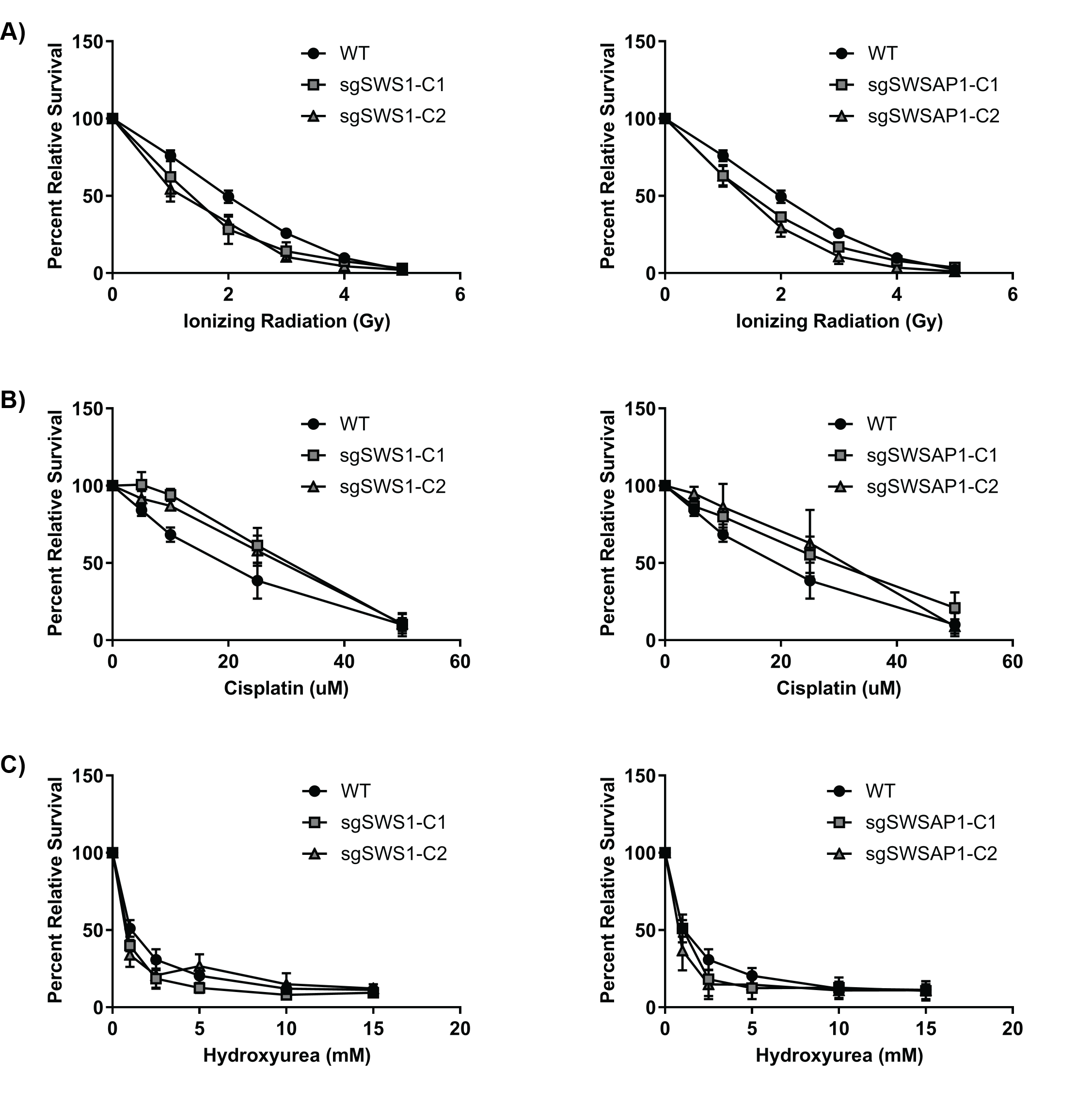


**Figure S2. Sensitivity of WT, sgSWS1-C1, sgSWS1-C2, sgSWSAP1-C1, and sgSWSAP1-C2 RPE-1 cells to DNA damaging agents.** Clonogenic survival assays using the indicated cell lines were treated with the listed agent at the indicated concentrations, seeded at colony forming density and stained with crystal violet 8 days post-seeding. Mean percent survival relative to parental RPE-1 cells (WT) and standard errors of the means are shown. All experiments were done in triplicate. Statistical differences were calculated by fitting a non-linear regression model to each data set and comparing the slopes between parental RPE-1, sgSWS1-C1, sgSWS1-C2, sgSWSAP1-C1 and sgSWSAP1-C2 cells. **A)** Ionizing radiation (p=0.0756). **B)** Cisplatin (1 h treatment) (p=0.1545). **C)** Hydroxyurea (24 h treatment) (p=0.4060).

**Supplemental Figure 3**


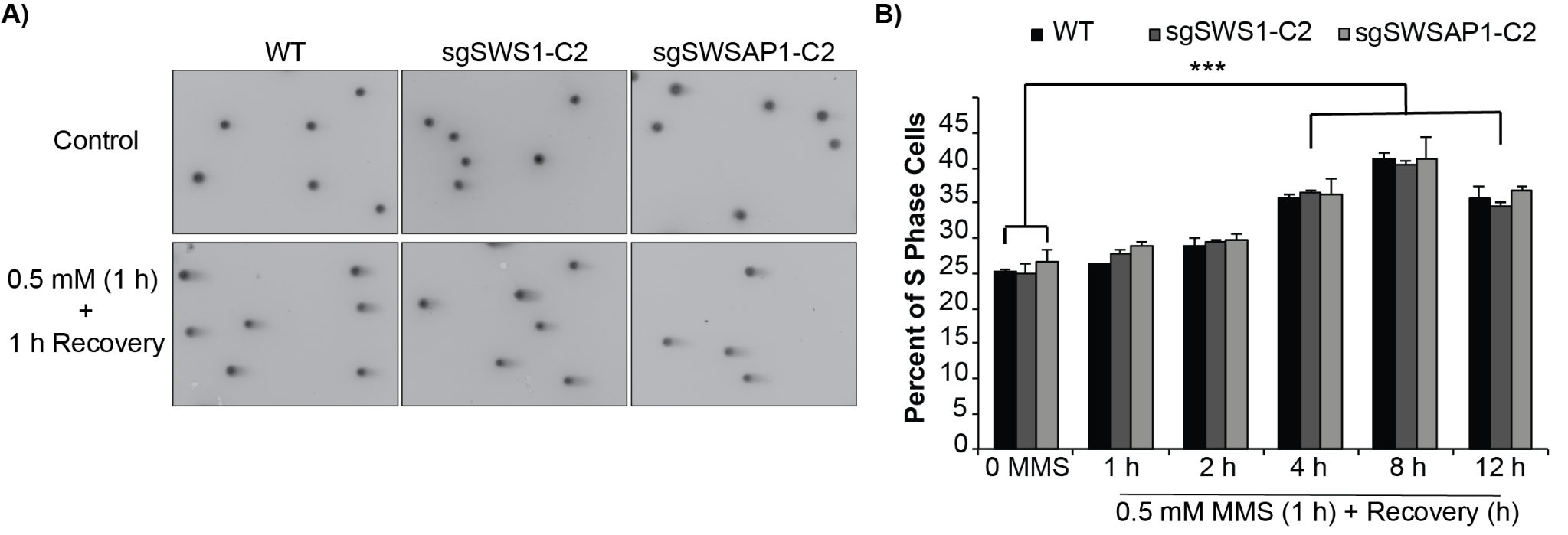


**Figure S3.** **MMS induces double-strand breaks, an S-phase delay and activates DNA damage checkpoint signaling in WT, sgSWS1-C2 and sgSWSAP1-C2 RPE-1 cells.** **A)** Representative images of the neutral comet assay in WT, sgSWS1-C2, and sgSWSAP1-C2 RPE-1 untreated cells and after exposure to 0.5 mM MMS (1 h) and 1 h recovery. **B)** Percent of S phase cells observed by FACS analysis in WT, sgSWS1-C2 and sgSWSAP1-C2 RPE-1 untreated and MMS-treated (0.5 mM for 1 hr) cells 1-12 h following MMS exposure. Mean percent of S phase cells and standard error of the mean are graphed. Experiments were done in duplicate. Statistical differences were calculated using two-way ANOVA, with treatment and cell lines as the two factors. MMS induced a statistically significant (***p<0.0005) S-phase arrest but there were no significant differences between cell lines.

**Supplemental Figure 4**


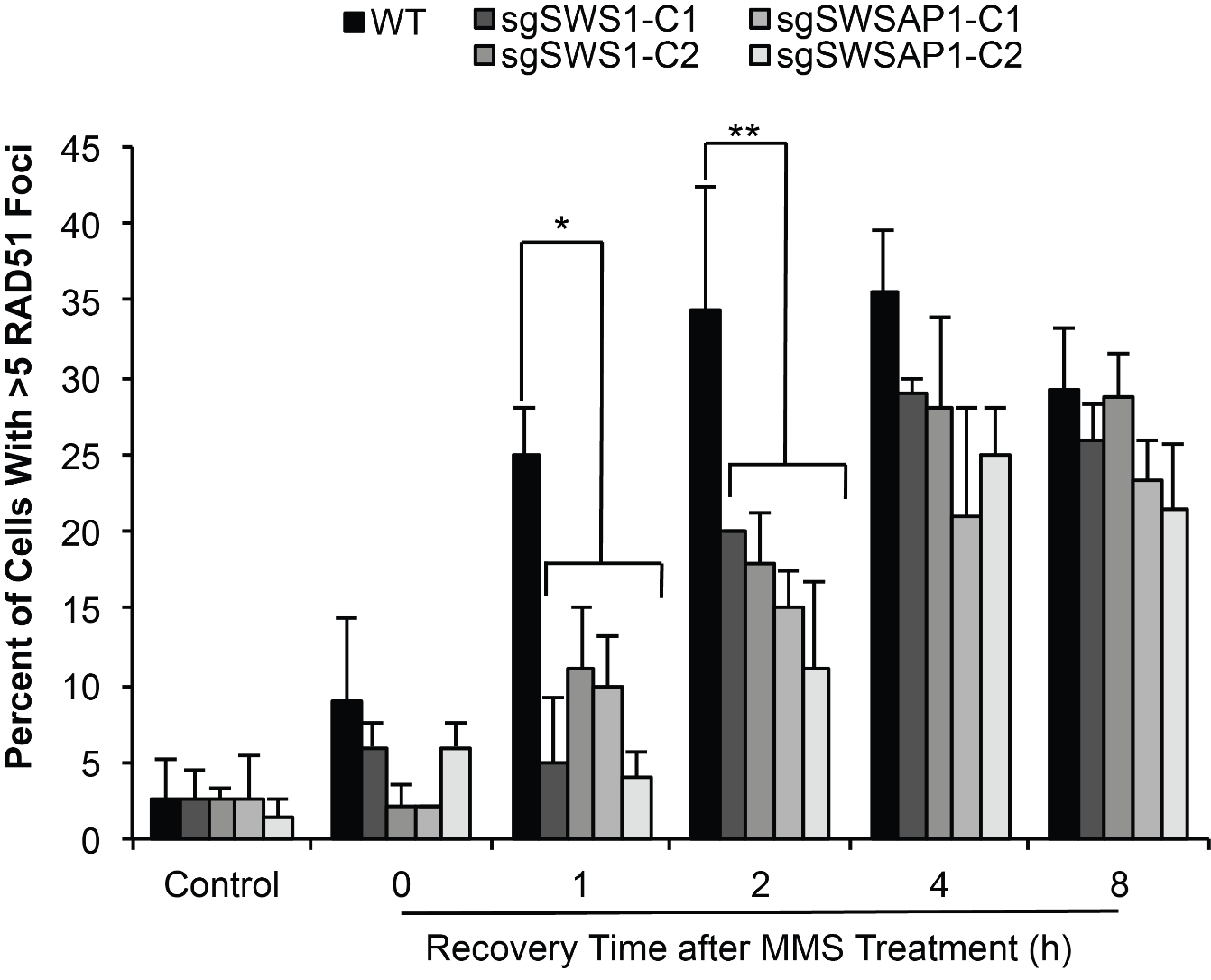


**Figure S4.** **The percent of** **sgSWS1 and sgSWSAP1 cells exhibiting >5 RAD51 foci is reduced after 1 and 2 h of MMS exposure.** Percent of WT, sgSWS1-C1, sgSWS1-C2, sgSWSAP1-C2, and sgSWSAP1-C2 RPE-1 cells with >5 RAD51 foci after exposure to 0.5 mM MMS (1 h) and 0-8 h recovery. Mean and standard error of the mean of 3 independent experiments are shown. Statistical differences were calculated using two-way ANOVA, with treatment and cell lines as the two factors. Both sgSWS1 and sgSWSAP1 cell lines exhibited a statistically significant (*p<0.05 and **p<0.005) reduction in the percent of cells with >5 RAD51 foci than parental RPE-1 cells at the indicated time points.

**Supplemental Figure 5**


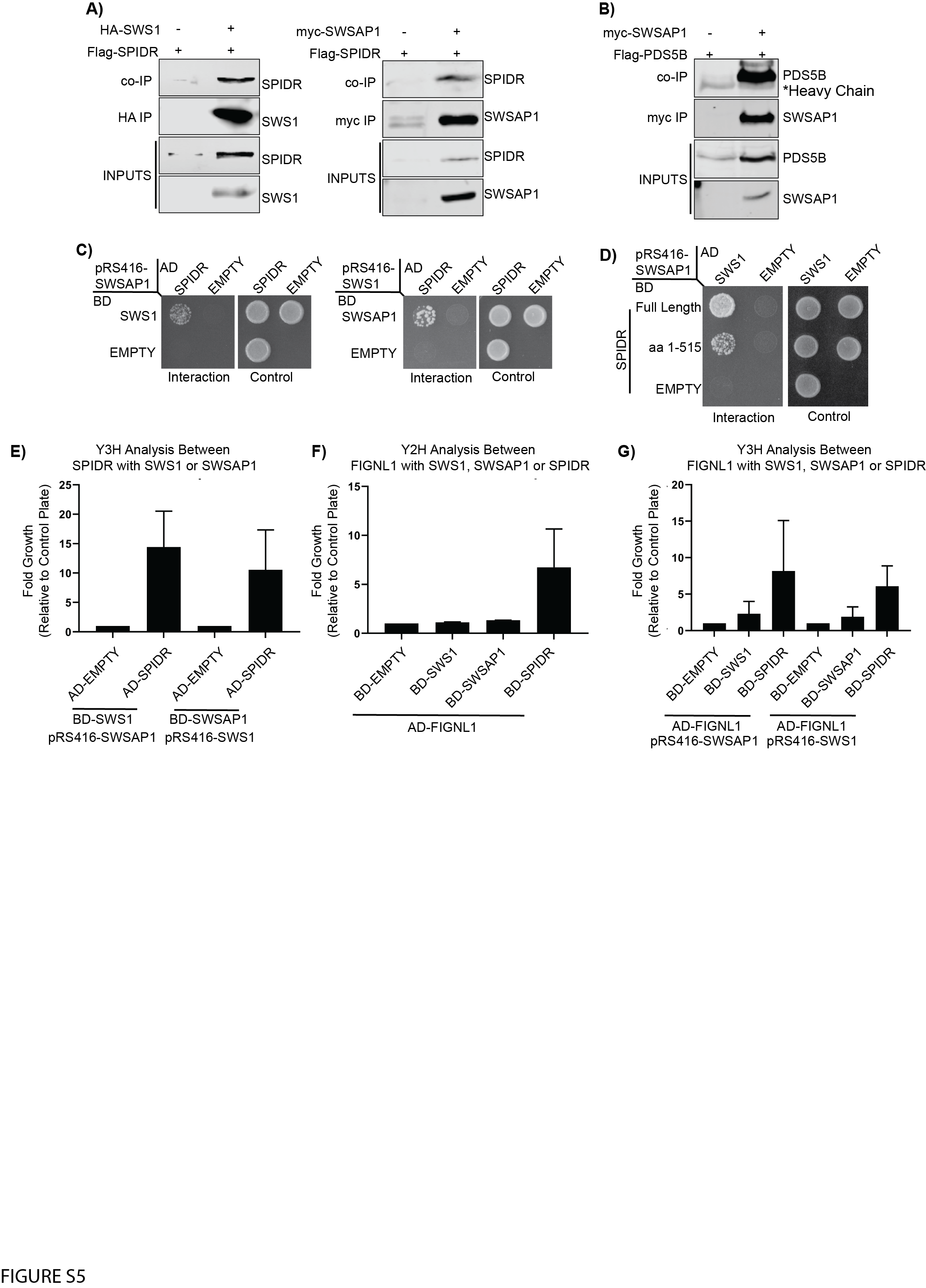


**Figure S5. SWS1 and SWSAP1 interact with SPIDR by co-immunoprecipitation and yeast-three-hybrid. A)** RPE-1 cells were transiently transfected with Flag-SPIDR and either HA-SWS1 or myc-SWSAP1. HA-SWS1 or myc-SWSAP1 were immunoprecipitated using anti-HA- or anti-myc-conjugated beads (HA IP and myc IP, respectively) and western blotted for SPIDR (co-IP) using the Flag antibody. Inputs represent 10% of the protein lysate. **B)** Co-immunoprecipitations of SWSAP1 and an N-terminal PDS5B fragment (a.a. 1-529) in RPE-1 cells. RPE-1 cells were transiently transfected with myc-SWSAP1 and Flag-N-term-PDS5B. Myc-SWSAP1 was immunoprecipitated using myc-conjugated beads. The N-terminal PDS5B fragment was co-immunoprecipitated by myc-SWSAP1. **C)** The PJ694a yeast strain was transformed with three plasmids; 1) a plasmid where SPIDR was fused to the GAL4-DNA activating domain (AD; pGAD-SPIDR), 2) a plasmid where either SWSAP1 or SWS1 was fused to the GAL4-DNA binding domain (BD; pGBD-SWSAP1 or pGBD-SWS1), and 3) a plasmid that constitutively expressed either SWS1 or SWSAP1 (pRS416-SWS1, pRS416-SWSAP1). A yeast-three-hybrid interaction between SPIDR and SWSAP1 or SWS1 was assayed by plating the yeast on SC-LEU-TRP-URA-HIS (Interaction, indicated by growth) and compared to the loading control SC-LEU-TRP-URA (Control). **D)** The PJ694a yeast strain was transformed with three plasmids; 1) a plasmid where SWS1 was fused to the GAL4-DNA activating domain (AD; pGAD-SWS1, 2) a plasmid where full length SPIDR or a SPIDR truncation (amino acids 1-515) was fused to the GAL4-DNA binding domain (BD; full length, aa 1-515), and 3) a plasmid that constitutively expresses SWSAP1 (pRS416-SWSAP1). A yeast-three-hybrid interaction between SWS1 with the full length or the aa 1-515 SPIDR fragment was assayed by plating the yeast on SC-LEU-TRP-URA-HIS (Interaction, indicated by growth) and compared to the loading control SC-LEU-TRP-URA (Control). **E)** Quantitative analysis of Y3H between SPIDR with SWS1 or SWSAP1. Data are shown as average growth (relative to control plate) ± standard deviation. Data are from three independent experiments. **F)** Quantitative analysis of Y2H between FIGNL1 with SWS1, SWSAP1 or SPIDR. Data are shown as average growth (relative to control plate) ± standard deviation. Data are from two independent experiments. **G)** Quantitative analysis of Y3H between FIGNL1 with SWS1, SWSAP1 or SPIDR. Data are shown as average growth (relative to control plate) ± standard deviation. Data are from two independent experiments.

**Supplemental Figure 6**


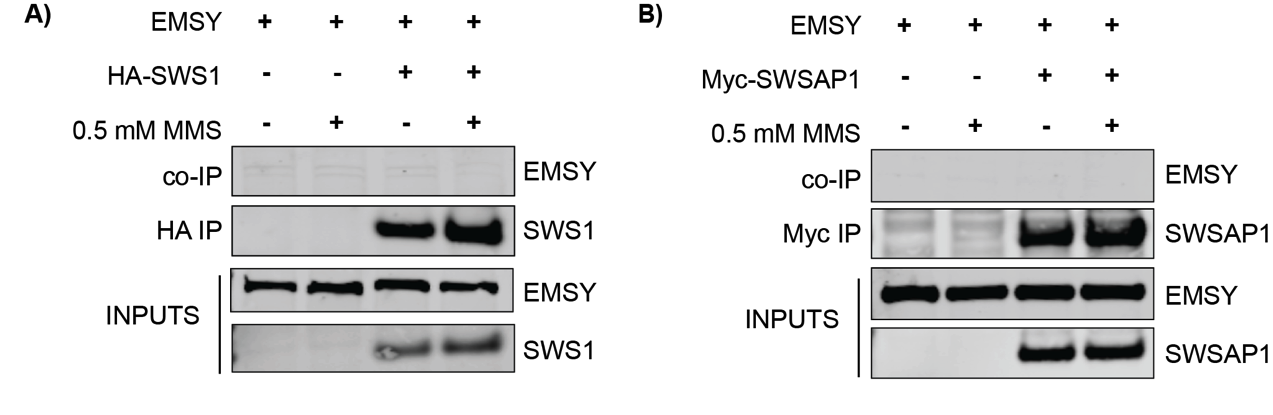


**Figure S6. SWS1 and SWSAP1 do not co-immunoprecipitate EMSY.** **A)** RPE-1 cells were transiently transfected with HA-SWS1 and an untagged EMSY construct. HA-SWS1 was immunoprecipitated from untreated and MMS-treated cells (0.5 mM MMS for 1 h and 4 h recovery) using anti-HA-conjugated beads (HA IP) and western blotted for EMSY (co-IP) using an anti-EMSY antibody. Inputs represent 10% of the protein lysate. **B)** RPE-1 cells were transiently transfected with Myc-SWSAP1 and an untagged EMSY construct. Myc-SWSAP1 was immunoprecipitated from untreated and MMS-treated cells (0.5 mM MMS for 1 h and 4 h recovery) using anti-Myc-conjugated beads (Myc IP) and western blotted for EMSY (co-IP) using an anti-EMSY antibody. Inputs represent 10% of the protein lysate.
